# Effects of BSF on Podocyte Apoptosis via Regulating the ROS-Mediated PI3K/AKT Pathway in DN

**DOI:** 10.1155/2019/9512406

**Published:** 2019-12-07

**Authors:** Fang-qiang Cui, Yue-Fen Wang, Yan-bin Gao, Yuan Meng, Zhen Cai, Cun Shen, Zhi-qiang Liu, Xin-can Jiang, Wen-jing Zhao

**Affiliations:** ^1^Department of Nephrology, Beijing Hospital of Traditional Chinese Medicine, Capital Medical University, 23 Meishuguanhou Street, Dongcheng District, Beijing 100010, China; ^2^School of Traditional Chinese Medicine, Capital Medical University, No. 10, Youanmenwai, Xitoutiao, Fengtai District, Beijing 100069, China; ^3^Beijing Key Lab of TCM Collateral Disease Theory Research, No. 10, Youanmenwai, Xitoutiao, Fengtai District, Beijing 100069, China

## Abstract

Diabetic nephropathy (DN) is the leading cause of end-stage renal disease (ESRD). The ROS-mediated PI3K/AKT pathway plays a key role in podocyte apoptosis and DN progression. Our previous study demonstrated that Baoshenfang (BSF) can decrease proteinuria and attenuate podocyte injury. However, the effects of BSF on podocyte apoptosis induced by the ROS-mediated PI3K/AKT pathway remain unclear. Herein, in vivo and in vitro studies have been performed. In our in vivo study, BSF significantly decreased 24-h urinary protein, serum creatinine, and blood urea nitrogen levels in DN mice. Meanwhile, BSF significantly inhibited oxidative stress and podocyte apoptosis in our in vivo and in vitro studies. Moreover, BSF significantly decreased the inhibition of the PI3K/AKT pathway induced by HG in DN. More importantly, the effects of BSF on podocyte apoptosis were reversed by PI3K siRNA transfection. In conclusion, BSF can decrease proteinuria and podocyte apoptosis in DN, in part through regulating the ROS-mediated PI3K/AKT pathway.

## 1. Introduction

Diabetic nephropathy (DN), a common and serious microvascular complication of diabetes mellitus (DM), is the leading cause of end-stage renal disease (ESRD) [1]. It has been demonstrated that many factors are involved in the progression of DN. However, the exact mechanisms underlying DN are unclear. Previous studies have found that the number of podocytes was significantly decreased in DN, which has been demonstrated by previous studies [2–4]. Podocyte apoptosis mainly accounting for the decreased number of podocytes [5]. This finding suggests that podocyte apoptosis is the main pathomechanism of DN [6, 7].

ROS-mediated PI3K/AKT is an important pathway for regulating podocyte apoptosis in DN [8–10]. It has been demonstrated that HG (high glucose) can increase the levels of reactive oxygen species (ROS) and induce oxidative stress in podocytes in DN [11]. Meanwhile, ROS decreases PI3K expression and inhibits AKT phosphorylation [12, 13]. Decreased AKT phosphorylation increases Bax and caspase-3 expressions and induces podocyte apoptosis [14]. Thus, regulating the ROS-mediated PI3K/AKT pathway in podocytes may be an important potential targeted therapy for DN in the future.

BSF, as a common TCM compound, has been widely used in the treatment of DN in our clinical practice. BSF consists of a group herbal medicines including *Astragalus membranaceus*, *Rehmannia*, *Salvia miltiorrhiza Bunge*, *Cuscuta chinensis*, *Herba Artemisiae Anomalae*, *Euonymusalatus*, and *Hirudo*. Liquid chromatography-mass spectrometry (LC-MS) has been performed for analyzing the main substances in BSF in our previous study [15]. Moreover, we found that BSF significantly decreased 24-hour urinary protein, serum creatinine, and blood urea nitrogen levels in DN patients. More importantly, BSF significantly inhibited podocyte apoptosis in diabetic rats. However, the effects of BSF on ROS-mediated PI3K/AKT pathway are unclear.

## 2. Methods

### 2.1. Animals

Our animal experiments were performed in accordance with the National Institutes of Health guide. All mice in our study were purchased from the Chinese Academy of Medical Sciences (Beijing, China). Our animal study consisted of the following three groups: normal control (NC) group, diabetic nephropathy (DN) group, and BSF group. Twenty KK-Ay mice (male, 8 weeks, and weighing 30-40 g) were randomly divided into the DN and BSF groups. Ten C57BL/6J mice (male, 8 weeks, and weighing 30-40 g) were fed as the NC group. All KK-Ay mice were fed with high-fat fodder for 4 weeks, while C57BL/6J mice were fed with regular fodder. Afterwards, the 24-h proteinuria was detected. The levels of 24-h proteinuria were significantly increased in KK-Ay mice compared with C57BL/6J mice. Then, treatment was started. Mice in the BSF group were gavaged with 0.75 g/kg/d BSF solution and mice in the NC and DN groups were gavaged with normal saline at an equal volume. The treatment lasted for 12 weeks. Afterwards, the serum and renal cortex of all the mice were collected for the purpose of this study.

### 2.2. Preparation of Rat Serum Containing Drug

SD rats were purchased from the Chinese Academy of Medical Sciences (Beijing, China). All SD rats (male, 8 weeks, and weighing 440 g to 460 g) were randomly divided into the following four groups: NC group, low-dose BSF group, medium-dose BSF group, and high-dose BSF group. Rats in the BSF groups were gavaged with BSF solution at a dose of 1 g, 2 g, or 4 g/ml per day for three days. Rats in the NC group were gavaged with an equal volume of normal saline as a control. Afterwards, serum was collected and isolated. The isolated serum was then placed in a water bath for 30 min at 56°C. The serum-containing drug was finally stored at −80°C.

### 2.3. Cell Culture and Treatment

A conditionally immortalized mouse podocyte line was used in our in vitro study. Podocytes were obtained from the national platform of experimental cell resources for science and technology. Podocytes were cultured in DMEM/low glucose (HyClone, Logan, UT, United States) with IFN-*γ* (PeproTech, Rocky Hill, New Jersey, USA) at 33°C for proliferation. Podocytes were then cultured at 37°C without IFN-*γ* for differentiation. Upon reaching 80% confluence, the podocytes were divided into the following four groups: normal control group (NC group), high-glucose group (HG group), BSF group, and PI3K siRNA group. Podocytes from the NC group were treated with DMEM containing 5.5 mmol/L glucose+normal rat serum. Podocytes from the HG group were treated with DMEM containing 5.5 mmol/L glucose+24.5 mmol/L glucose+normal rat serum. Podocytes from the BSF group were treated with medium containing 5.5 mmol/L glucose+24.5 mmol/L glucose+ rat serum with BSF. Podocytes from the PI3K siRNA group were cultured in medium containing 5.5 mmol/L glucose+24.5 mmol/L glucose+rat serum with BSF+PI3K siRNA. All of the treatments lasted for 24 h. The podocytes were then collected for the purpose of these experiments.

### 2.4. PI3K siRNA Transfection

The PI3K siRNA was provided by Santa Cruz Biotechnology (Santa Cruz, CA, USA). The Lipofectamine 2000 transfection reagent (Invitrogen) was used for PI3K siRNA transfection according to the manufacturer's protocol. Briefly, podocyte was cultured in 24-well plates. When the cells reached 60–70% confluence, Lipofectamine 2000/siRNA complexes were added to the podocyte. After incubation for 6 h at 37°C, the mixture was replaced with DMEM supplemented with 10% FBS and incubation was continued for 2 days. To confirm the transfection, PI3K expression was detected by Western blot and RT-PCR.

### 2.5. CCK-8

Podocytes were cultured in a 96-well plate. Upon reaching 10000 cells/well, the podocytes were treated with different media according to their groups. The treatment lasted for 24 h. CCK-8 solution (10 *μ*l) was then added to each well. The podocytes were incubated with CCK-8 solution at 37°C for 2 h. The optical density (OD) of each well was detected by a spectrophotometer at 450 nm.

### 2.6. He, Masson, and PAS Staining

Renal tissues were fixed with 4% paraformaldehyde solution and embedded in paraffin. The renal tissues were then sectioned to 4 *μ*m slices. The slices were dewaxed by dimethylbenzene. After that, the tissues slices were stained by hematoxylin-eosin (HE), periodic acid-Schiff (PAS), and Masson's trichrome. Slices were then dehydrated to transparency and mounted by a neutral gum. The stained tissues were observed with a light microscope equipped with a camera.

### 2.7. Western Blot

The renal cortex tissues and collected podocytes were lysed with lysis buffer. Equal amounts of protein extracted from the lysed tissues were separated by electrophoresis. Then, the separated proteins were transferred to polyvinylidene difluoride membranes. After blocking with 5% nonfat dry milk, the polyvinylidene difluoride membranes were incubated with primary antibodies overnight at 4°C. The peroxidase secondary antibodies were then added to the membranes for incubation at room temperature for 1 h. The blots were visualized with LumiGLO reagent and peroxide, followed by exposure to an X-ray film. Western blot analyses were performed at least in triplicate. The following antibodies and dilutions were used: anti-NOX-4 antibody (Abcam, UK, Ab109225, 1 : 1000), anti-PI3K antibody (Abcam, UK, ab151549, 1 : 1000), anti-P-PI3K antibody (Abcam, UK, ab140307, 1 : 500), anti-AKT antibody (Abcam, UK, ab179463, 1 : 500), anti-P-AKT antibody (Abcam, UK, ab38449, 1 : 1000), anti-Bax antibody (Abcam, UK, ab182737, 1 : 1000), anti-Bcl-2 antibody (Abcam, UK, ab692, 1 : 1000), anti-caspase-9 antibody (Abcam, UK, ab202068, 1 : 500), and anti-caspase-3 antibody (Abcam, UK, ab13847, 1 : 500).

### 2.8. RT-PCR

The TRIzol reagent (Invitrogen, Carlsbad, CA, USA) was used for total RNA isolation. The isolated RNA was then reverse-transcribed into cDNAs. Then, RT-PCR was performed using SYBR green real-time quantitative reverse transcription PCR (qRT-PCR) (Applied Biosystems), and the relative mRNA levels were calculated by the 2^−ΔΔCt^ method. The primer sequences are as follows: mouse NOX-4, TGCTACTGCCTCCATCAAGTCAAG (forward primer) and ACTCCAATGCCTCCAGCCACA (reverse primer); mouse Bax, ATGCGTCCACCAAGAAGCTGA (forward primer) and AGCAATCATCCTCTGCAGCTCC (reverse primer); and mouse Bcl-2, CCGGGAGAACAGGGTATGATAA (forward primer) and CCCACTCGTAGCCCCTCTG (reverse primer).

### 2.9. Immunohistochemistry

Renal tissues were fixed with 4% paraformaldehyde solution and embedded in paraffin. The renal tissues were then sectioned to 4 *μ*m. After antigen retrieval, the sectioned tissues were incubated with primary antibodies at 4°C for 24 h. Species-specific secondary antibodies and diaminobenzidine were added to the sectioned tissues, followed by incubation for 1 h at room temperature. The sections were observed with a fluorescence microscope.

### 2.10. Immunofluorescence

Podocytes were cultured in a 12-well plate. Upon reaching 80% confluence, the podocytes were treated with different media for 24 h. Then, the podocytes were fixed with 4% paraformaldehyde for 30 min. After blocking with 2.5% dunk serum, the podocytes were incubated with primary antibodies at 4°C overnight. Next, secondary antibodies were added to the cultured podocytes, followed by incubation for 2 h at room temperature. After counterstaining with DAPI, the cells were observed under a confocal microscope (Leica TCS SP5 MP, Leica, Heidelberg, Germany).

### 2.11. Phalloidin Staining

Podocytes were cultured in a 12-well plate. Upon reaching 80% confluence, the podocytes were treated with different media for 24 h. Then, the podocytes were fixed with 4% paraformaldehyde for 30 min. The podocytes were then incubated with phalloidin for 30 min at room temperature. After counterstaining with DAPI, the cells were observed under a confocal microscope (Leica TCS SP5 MP, Leica, Heidelberg, Germany).

### 2.12. ROS Detection

Podocytes were cultured in a 24-well plate. Upon reaching 80% confluence, the podocytes were treated with different media for 24 h. ROS detection was performed using an ROS detection kit (Beyotime, Haimen, China) according to the manufacturer's protocol. The podocytes were incubated with 10 *μ*M DCFH-DA for 20 min at 37°C. The fluorescence intensity was observed and recorded by a fluorescence microscope.

### 2.13. TUNEL

TUNEL detection was performed using a TUNEL detection kit (Nanjing Jiancheng Bioengineering Institute, Nanjing, China). Renal tissue sections were incubated with protease K at 37°C for 30 min. After washing with 3% H_2_O_2_, the renal tissues were incubated with a mixture of terminal deoxynucleotidyl transferase (TdT) and biotin-dUTP. Streptavidin-horseradish peroxidase was then added to the renal tissue sections. Then, the renal tissue sections were stained with DAB and observed using a fluorescence microscope.

### 2.14. Flow Cytometry Analysis

Podocytes were collected by centrifugation at 2000 rpm for 5 min. The collected podocytes were washed with cold PBS and then resuspended in binding buffer. Next, the podocytes were stained with Annexin V-FITC and PI for 15 min. Cell apoptotic data were then analyzed using FACScan.

### 2.15. Statistical Analysis

Data from our study are presented as the mean ± SEM. Statistical analyses were performed by one-way ANOVA. Then, the Bonferroni multiple comparison test (for comparisons of more than 2 groups) or Student's *t* test (for comparisons of 2 groups) were performed. *P* < 0.05 was considered statistically significant.

## 3. Results

### 3.1. Effects of BSF on Renal Function, Renal Pathology, and Podocyte Injury in DN Mice

**Figure 1:**
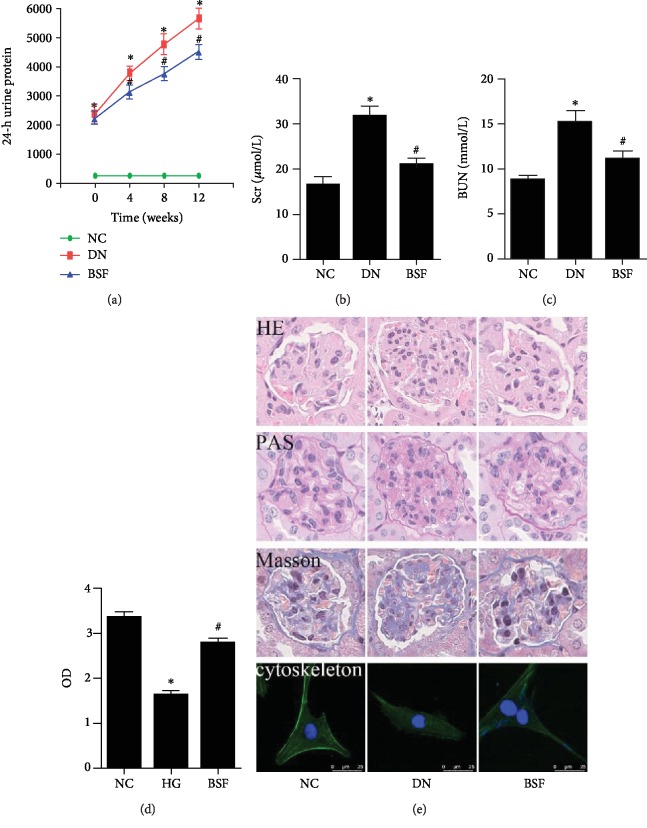
Effects of BSF on renal function, renal pathology, and podocyte injury in DN mice. (a) Comparison of 24-h proteinuria from mice in different groups. (b) Comparison of Scr from mice in different groups. (c) Comparison of BUN from mice in different groups. (d) Comparison of OD from cultured podocytes in different groups detected by CCK-8. (e) Representative photograph of HE, PAS, Masson, and phalloidin staining in different groups.

The 24-h proteinuria, serum creatinine, blood urea nitrogen, and renal pathology were detected in our study. Our results showed that the 24-h proteinuria, serum creatinine, and blood urea nitrogen of DN group were significantly increased. Compared with the DN group, 24-h proteinuria, serum creatinine, and blood urea nitrogen levels of BSF group were significantly decreased (Figures 1(a)–1(c)). Meanwhile, mesangial matrix of mice in DN group was significantly accumulated. BSF significantly decreased the accumulation of mesangial matrix in DN (Figure 1(e)). CCK-8 detection was performed for detecting podocyte viability of different groups in our in vitro study. Our results showed that HG significantly decreased the cell viability of cultured podocytes. Serum-containing BSF significantly increased podocyte viability induced by HG (Figure 1(d)). Moreover, the podocyte cytoskeleton was observed in our in vitro study. The podocyte cytoskeleton of NC group was observed as parallel bundles of stress fibers. Intracellular actin stress fibers were abolished and replaced by a cortical actin web, resulting in a polygonal cellular shape in the HG group. BSF resumed intracellular actin stress fiber production and maintained the normal cellular shape induced by HG (Figure 1(e)).

### 3.2. Effects of BSF on Podocyte Apoptosis in DN Mice and HG-Cultured Podocytes

**Figure 2:**
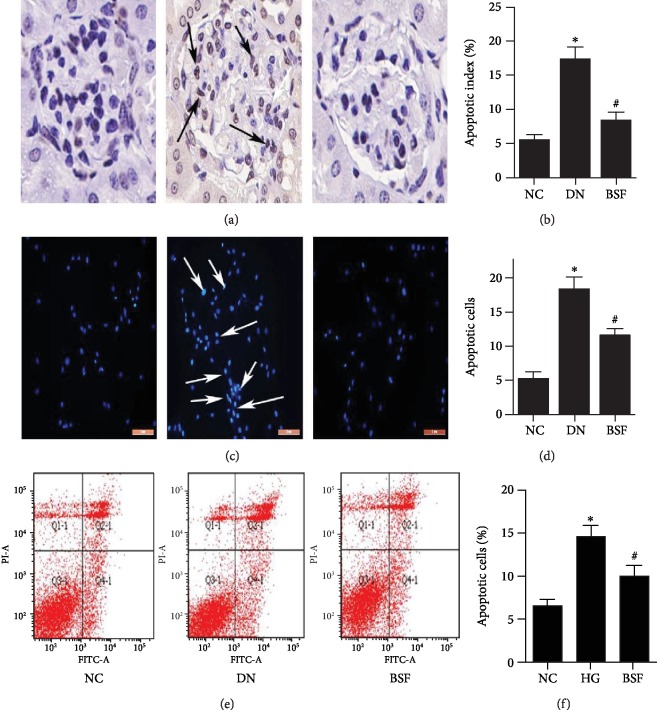
Effects of BSF on podocyte apoptosis in DN mice and HG-cultured podocytes. (a) Representative photograph of TUNEL staining (arrows indicate apoptotic podocytes). (b) Comparison of apoptotic cells in glomerulus from different groups detected by TUNEL. (c) Representative photograph of Hoechst 33258 staining (arrows indicate apoptotic podocytes). (d) Comparison of apoptotic cells in cultured podocytes from different groups detected by Hoechst 33258. (e) Representative photograph of flow cytometry analysis. (f) Comparison of apoptotic cells among cultured podocytes from different groups detected by flow cytometry.

Podocyte apoptosis was detected by multiple methods in our in vivo and in vitro studies. Renal tissue podocyte apoptosis in DN mice was observed by TUNEL. Our results showed that podocyte apoptosis was significantly increased in DN mice. Compared with the DN group, podocyte apoptosis of mice in BSF group was significantly decreased (Figures 2(a) and 2(b)). Podocyte apoptosis was also detected by Hoechst 33258 and HITC/PI staining in our in vitro study. HG significantly increased podocyte apoptosis among cultured podocytes. Compared with the HG group, podocyte apoptosis of BSF group was significantly decreased (Figures 2(c)–2(f)).

### 3.3. Effects of BSF on the ROS-Mediated PI3K/AKT Pathway in DN Mice and HG-Cultured Podocytes

**Figure 3:**
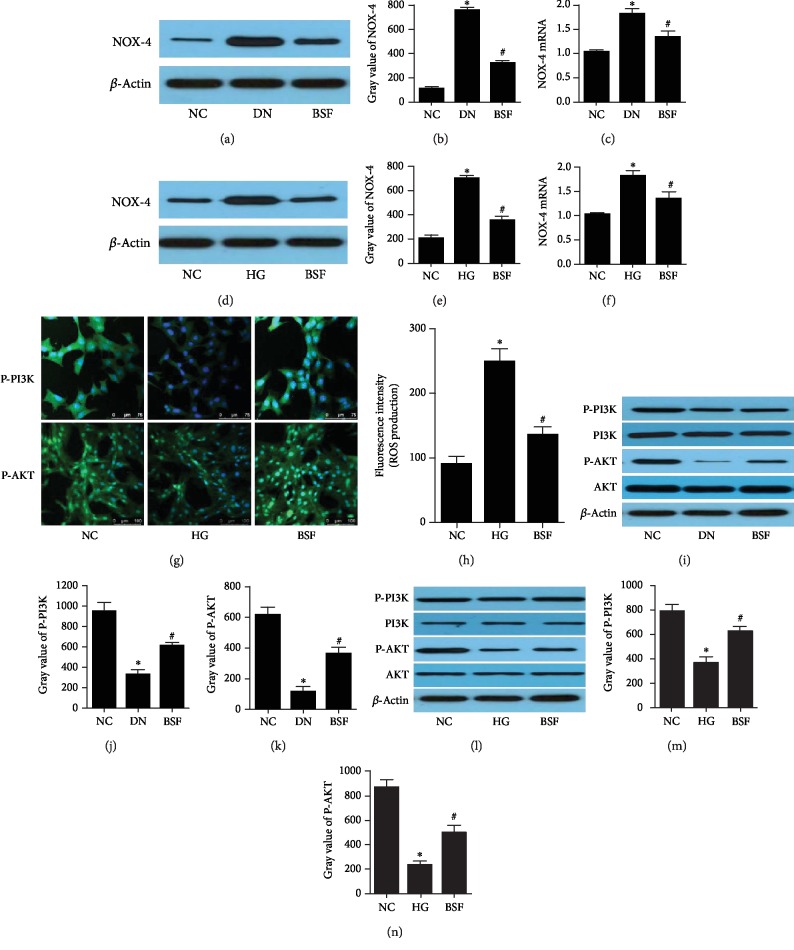
Effects of BSF on the ROS-mediated PI3K/AKT pathway in DN mice and HG-cultured podocytes. (a) Representative NOX-4 protein band in the renal cortex from mice in different groups. (b) Comparison of NOX-4 protein in the mouse renal cortex (*n* = 3). (c) Comparison of NOX-4 mRNA levels in the rat renal cortex (*n* = 3). (d) Representative NOX-4 protein bands from cultured podocytes in different groups. (e) Comparison of NOX-4 protein from cultured podocytes (*n* = 3). (f) Comparison of NOX-4 mRNA levels in cultured podocytes (*n* = 3). (g) Representative photograph of PI3K and P-AKT staining in cultured podocytes. (h) Comparison of ROS production in cultured podocytes from different groups. (i) Representative PI3K, P-PI3K, AKT, and P-AKT protein bands in the renal cortex of mice in different groups. (j) Comparison of P-PI3K protein in the mouse renal cortex (*n* = 3). (k) Comparison of P-AKT protein in the mouse renal cortex (*n* = 3). (l) Representative PI3K, P-PI3K, AKT, and P-AKT protein bands in cultured podocytes in different groups. (m) Comparison of P-PI3K protein in cultured podocytes (*n* = 3). (n) Comparison of P-AKT protein in cultured podocytes (*n* = 3).

NOX-4 expression was detected in our in vivo and in vitro studies. NOX-4 expression was significantly increased in DN mice and HG-cultured podocytes. Moreover, BSF significantly decreased NOX-4 expression of podocytes in DN (Figures 3(a)–3(f)). Meanwhile, ROS production of podocyte in HG group was significantly increased. BSF significantly decreased ROS production of podocyte induced by HG (Figure 3(h)). To explore the effects of BSF on the PI3K/AKT pathway, PI3K, P-PI3K, AKT, and P-AKT expressions were detected. Our results showed that HG significantly decreased P-PI3K and P-AKT expressions in our in vivo and in vitro studies and BSF significantly increased P-PI3K and P-AKT expressions in podocytes induced by HG (Figures 3(g) and 3(i)–3(n)).

### 3.4. Effects of BSF on Bax, Bcl-2, Caspase-9, and Caspase-3 Expressions in DN Mice and HG-Cultured Podocytes

**Figure 4:**
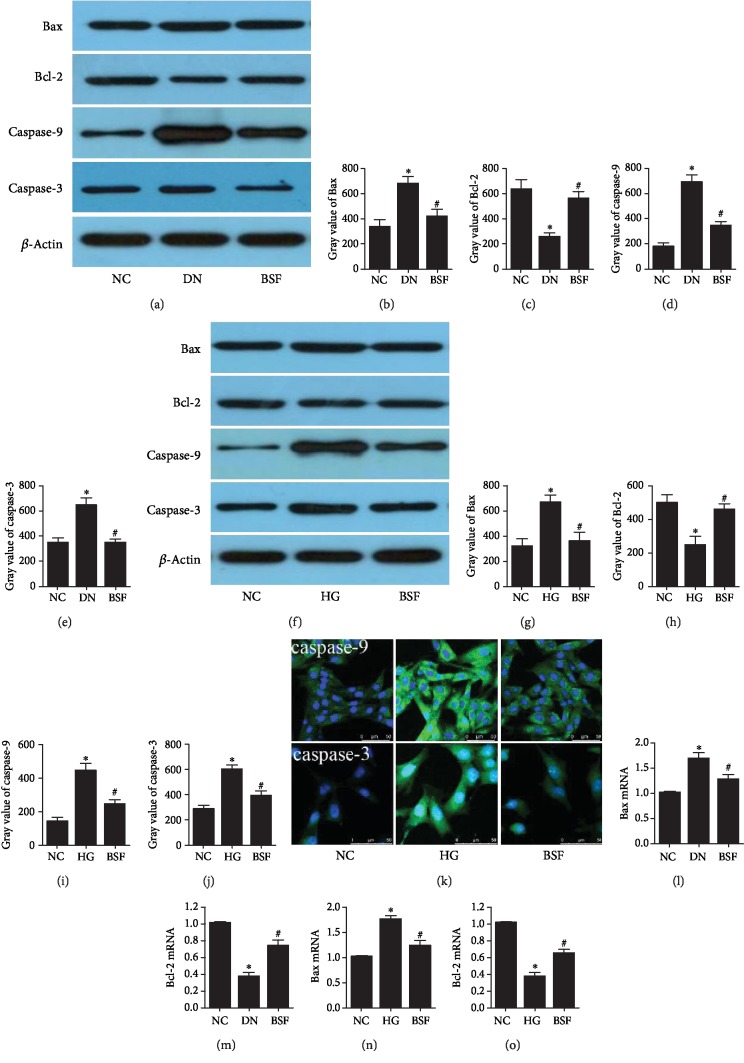
Effects of BSF on Bax, Bcl-2, caspase-9, and caspase-3 expressions in DN mice and HG-cultured podocytes. (a) Representative Bax, Bcl-2, caspase-9, and caspase-3 protein bands in the renal cortex from mice in different groups. (b) Comparison of Bax protein in the mouse renal cortex (*n* = 3). (c) Comparison of Bcl-2 protein in the mouse renal cortex (*n* = 3). (d) Comparison of caspase-9 protein in the mouse renal cortex (*n* = 3). (e) Comparison of caspase-3 protein in the mouse renal cortex (*n* = 3). (f) Representative Bax, Bcl-2, caspase-9, and caspase-3 protein bands in cultured podocytes in different groups. (g) Comparison of Bax protein in cultured podocytes (*n* = 3). (h) Comparison of Bcl-2 protein in cultured podocytes (*n* = 3). (i) Comparison of caspase-9 protein in cultured podocytes (*n* = 3). (j) Comparison of caspase-3 protein in cultured podocytes (*n* = 3). (k) Representative photograph of caspase-9 and caspase-3 staining in cultured podocytes. (l) Comparison of Bax mRNA levels in the rat renal cortex (*n* = 3). (m) Comparison of Bcl-2 mRNA levels in the rat renal cortex (*n* = 3). (n) Comparison Bax mRNA levels in cultured podocytes (*n* = 3). (o) Comparison of Bcl-2 mRNA levels in cultured podocytes (*n* = 3).

Bax and Bcl-2 expressions were detected in our study. The results showed that HG significantly increased Bax expression and decreased Bcl-2 expression of podocyte in DN and HG group. BSF significantly decreased Bax expression and increased Bcl-2 expression induced by HG in DN (Figures 4(a)–4(c), 4(f)–4(h), and 4(l)–4(o)). Moreover, caspase-9 and caspase-3 expressions were also detected. Caspase-9 and caspase-3 expressions were significantly increased in DN mice and HG-cultured podocytes. BSF significantly decreased caspase-9 and caspase-3 expressions induced by HG in DN (Figures 4(a), 4(d)–4(f), and 4(i)–4(k)).

### 3.5. Effects of PI3K siRNA on Podocyte Apoptosis in the BSF Group in HG-Cultured Podocytes

**Figure 5:**
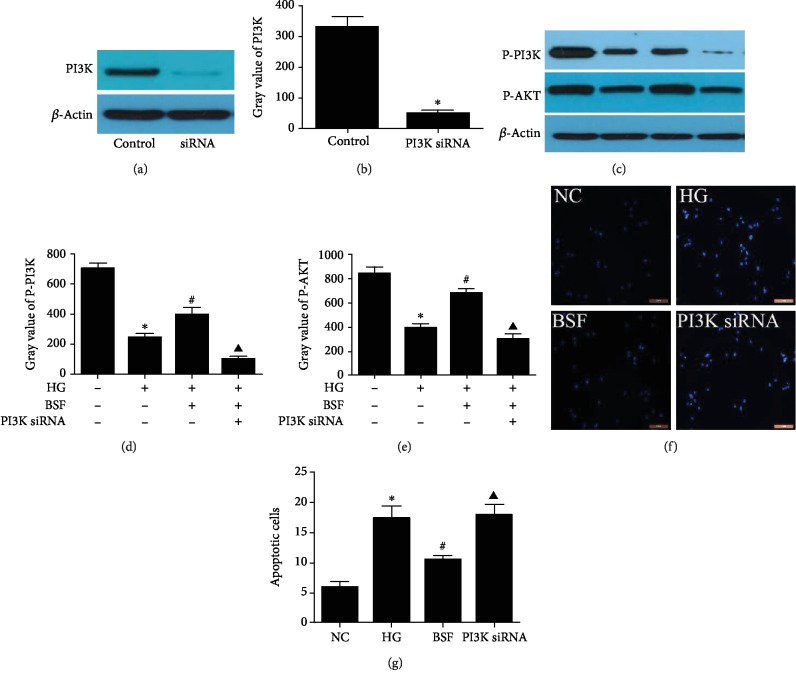
Effects of PI3K siRNA on podocyte apoptosis in the BSF group in HG-cultured podocytes. (a) Representative PI3K protein bands in cultured podocytes in different groups. (b) Comparison of PI3K protein in cultured podocytes (*n* = 3). (c) Representative P-PI3K and P-AKT protein bands in cultured podocytes in different groups. (d) Comparison of P-PI3K protein in cultured podocytes (*n* = 3). (e) Comparison of P-AKT protein in cultured podocytes (*n* = 3). (f) Representative photograph of Hoechst 33258 staining. (g) Comparison of apoptotic cells among cultured podocytes from different groups detected by Hoechst 33258.

To explore the relationship of podocyte apoptosis and the PI3K pathway, podocytes were transfected with PI3K siRNA. To confirm the transfection, PI3K protein expression was detected. Our results showed that PI3K protein expression was significantly decreased in siRNA group (Figures 5(a) and 5(b)). Moreover, P-PI3K, P-AKT, and podocyte apoptosis were detected after transfection. The increased P-AKT and P-PI3K expressions and decreased podocyte apoptosis induced by BSF were reversed by PI3K siRNA. (Figures 5(c)–5(g)).

## 4. Discussion

DN has been the leading cause of ESRD [1]. However, the exact mechanism underlying DN remains elusive. Podocytes, a kind of terminal differentiation cell, are the most important components of the glomerular filtration barrier (GBM) [16]. Previous studies have demonstrated that podocyte apoptosis is significantly increased in DN. Meanwhile, podocyte apoptosis plays a key role in proteinuria and the progression of DN [5]. Thus, and herein, podocyte apoptosis has been an important research focus of DN in recent years [17, 18]. However, treatment to attenuate podocyte apoptosis in DN is limited. Meanwhile, many clinical treatments have not effectively delayed the progression of DN. Thus, a new and effective treatment is urgently needed for DN patients. BSF has been widely used in the treatment of DN in our clinical practice. Moreover, BSF significantly decreased proteinuria and improved renal function in DN patients [15]. However, the molecular mechanism of BSF in treating DN is not completely understood.

BSF, a TCM compound, consists of a group of herbal medicines. Our previous study has analyzed the components of BSF by LC-MS. BSF is composed of 54 substances including quercetin, salvianolic acid B, luteolin, and astragaloside IV. It has been demonstrated that quercetin [19], salvianolic acid B [20], and luteolin [21] can decrease oxidative stress in many diseases. Moreover, astragaloside IV can significantly attenuate podocyte injury in DN [22]. In our study, the effects of BSF on proteinuria and renal function were explored in DN mice. Our study found that BSF significantly decreased proteinuria, serum creatinine, and blood urea nitrogen levels in DN mice. We also explored the effects of BSF on podocyte injury, podocyte apoptosis, cell viability, and actin cytoskeleton rearrangement in our in vivo and in vitro studies. Our study found that BSF significantly decreased podocyte apoptosis in DN mice and HG-cultured podocytes. Moreover, BSF significantly increased cell viability and inhibited actin cytoskeleton rearrangement in HG-cultured podocytes.

The role of oxidative stress in the progression of DN has been a focus in recent years [23, 24]. Oxidative stress can increase proteinuria and induce characterized abnormalities in the renal structure in DN [25]. Meanwhile, oxidative stress also plays a vital role in podocyte apoptosis [26]. Evidence suggests that HG can significantly increase ROS levels, and increased ROS can induce podocyte apoptosis [11]. BSF significantly decreased ROS production in high-glucose-cultured podocytes in our study. There are many ways to increase ROS production in podocytes. However, the activation of NADPH oxidase mainly accounts for the increased ROS production in podocytes [27]. Among the many NADPH oxidase members, NOX-4 oxidase plays a key role in oxidative stress and podocyte apoptosis in DN [28, 29]. Thus, the effects of BSF on NOX-4 expression in podocytes were explored in our in vivo and in vitro studies. Our studies found that BSF significantly decreased NOX-4 expression.

PI3K/AKT is an important pathway that regulates podocyte apoptosis [8–10]. The PI3K/AKT pathway can be inhibited by ROS in DN. Evidence suggests that ROS inhibits the phosphorylation of PI3K and AKT [12, 13]. To explore the effects of BSF on the PI3K/AKT pathway, PI3K, P-PI3K, AKT, and P-AKT were detected in our in vivo and in vitro studies. Our results showed that BSF significantly increased P-PI3K and P-AKT expressions and attenuated the inhibition of the PI3K/AKT pathway in DN. AKT, a key downstream effector of PI3K, plays a key role in podocyte apoptosis. Dephosphorylated AKT can increase Bax protein expression and decrease Bcl-2 expression. Bax is a proapoptotic protein and Bcl-2 is an inhibitor of apoptosis protein. The imbalance of Bax and Bcl-2 expressions activates caspase-9 and caspase-3 expressions, finally inducing podocyte apoptosis. Herein, Bax, Bcl-2, caspase-9, and caspase-3 expressions were detected in our study. BSF significantly decreased Bax, caspase-9, and caspase-3 expressions and significantly increased Bcl-2 expression in DN mice and high-glucose-cultured podocytes. Moreover, the effects of BSF on podocyte apoptosis were reversed by PI3K siRNA transfection.

In conclusion, BSF can decrease podocyte apoptosis and delay the progression of DN, partly through activating the PI3K/AKT pathway in podocytes.

## Data Availability

The data used to support the findings of this study are available from the corresponding author upon request.
